# Supplementary material for: Cortical oscillatory dysfunction in Parkinson disease during movement activation and inhibition
Source: PLoS One. 2022 Mar 4;17(3):e0257711. doi: 10.1371/journal.pone.0257711 (PMC8896690; doi:10.1371/journal.pone.0257711)
Supplement: Attachment — Submitted filename: Response to Reviewers.docx [file pone.0257711.s001.docx]

We would like to thank the reviewers for the time and effort that they invested in reviewing this manuscript. All comments have been carefully considered and included in the revised manuscript.

**Reviewer #1**:

**We understand the frustration expressed by Reviewer 1 that the manuscript is very difficult to follow. The data set is complex, including several trial types and response options with both the right and left hand, as well as temporal and spatial brain response data across multiple frequency bands. It has been challenging to sift through everything and distill it into a reader friendly format that captures all the findings. I would like to thank the reviewer again for his/her feedback, which is invaluable in clarifying the manuscript. Based on the reviewer’s comments we have added a summary of the results to the first paragraph of the discussion to make the material easier to digest. We have also edited the manuscript throughout for clarity, including removing background information that was not directly related**

*The description of the tasks is confusing. For instance, in the methods, it says that in the RB and LB task, the subjects must press an un-cued button but the task seems to be cued because it is called RB and LB. The authors should clarify this because they use the word clue for BR and BL but they consider the task un-cued.*

**The section describing the task, now called “*Activation Inhibition Task”* (starting on page 7, Line 157) has been revised to clarify the cued and un-cued/ activation vs. inhibition design.**

*In addition, there is no description in the methods on how the subjects enter their responses. It seems that they press a button with the right and/or left hand to choose their answers but this is not stated. In this context, results mention “right-hand trials” and left-hand trials” but the manuscript does not explain what this means.*

**In the original version, this information was contained in the last sentences of the (lengthy) description of the task. It has been moved to the first few sentences of the revised version, along with a more general description of the task: “Subjects were presented with a visual cue-target design task and required to respond to the target by pushing a button with one or both index fingers. Fiber optic button boxes (Photon Control, Inc. <http://www.photoncontrol.com/>) were held in both the left and right hands. Subjects were trained prior to entering the scanner to respond only to the target arrow(s) with their index fingers pressing hand-held button boxes.” (page 7 line 158)**

*This is all the more confusing because on page 12 and 13 of the results, it is stated that* *“right- and left-hand trials were similar, so data from right-hand response trials were used to illustrate the results. Right-hand results were chosen because all subjects were right-handed with right side dominant disease. Right- and left-hand response MEG data were not combined because sensory and motor cortex results were not aligned due to crossed inputs from the two hands, while frontal lobe activation was independent of response hand”.*

**To clarify this procedure we added the phrase “Right- and left-hand trials both tested activation and inhibition in a similar fashion, and results from right- and left-hand trials were similar, so data from right-hand response trials were used to illustrate the results. Right-hand results were chosen because all subjects were right-handed with right side dominant disease. This change, in combination with the clarification of the task description, should improve reader accessibility to the information.**

**Reviewer #2:** This is a very interesting study where the authors deeply discussed the cortical oscillatory dysfunction in early Parkinson's disease during movement activation and inhibition. I have some comments about this paper, and some suggestions too.

*In general, the manuscript sounds very technical and scientific. However, some clinical statements were not clear. For example, in the title the authors declare that in this study, the participants were in early stage of PD.*

*Can the authors please update the Hoehn and Yahr stages of which the participants were classified in their early stages?*

**The H & Y data has been added to table 1. In addition the word “early” has been removed from the title and the subject description.**

*I also suggest that the authors reduce the number of acronyms. Try to keep the acronyms to minimum.*

**We agree that there were too many acronyms. All acronyms were removed except for these relatively common ones:**

**ANOVA**

**EEG**

**fMRI**

**MRI**

**ROI**

**UPDRS**

**All acronyms are now spelled out the first time they are used.**

Introduction

*Page 3, lines 53-54 – Please, give a reference to the statement made.*

**On page 3, line 54 we have added references (Disbrow et al., 2013; Franz, 2006; Obeso et al., 2011) to the first sentence of the introduction for our statement “Response initiation and inhibition are functions fundamental to executive control that are disrupted in Parkinson disease (PD).”**

*Page 3, line 60 – What motor signs of PD are you referring? Only akinesia, as cited in line 61? Please, be clearer about this statement.*

**We clarified this point by qualifying the original statement:**

**“Furthermore, impairments in response activation and inhibition have been proposed to subserve some of the common motor signs of PD. For example, akinesia, or an inability to start movement [6–9] is consistent with a problem in movement activation [10].”**

**We also added another example: “Activation and inhibition are also associated with motor switching and sequencing, which are impaired in PD (Franz, 2006; Cools et al., 1984).” Page 3, Line 59-63.**

*Page 3, line 64 – Please state the “pre-movement external cueing” which are cited in the references 9-13. Such statement makes it easier for the reader.*

**Details have been added to this statement on page 3 lines 64-66: “However, deficits in response activation and inhibition associated with PD are complex and not fully understood. For instance, in PD, the latency to initiate movement can be shortened by the use of pre-movement external auditory or visual cueing such as walking with a metronome or use of floor markers (e.g., [12-16]).**

*Page 3, line 74 – I think the authors are referring to “Miller”, not colleagues here. If I am correct, please, change this.*

**This error has been corrected, the line, now on page 3 line 73 now reads: “Franz and Miller [23] found that people with mild to moderate PD demonstrated abnormal force output during movement activation of ‘Go’ responses, as well as problems inhibiting movement of ‘Nogo’ responses in comparison to controls, despite a lack of difference in mean reaction times between PD and control groups.**”

Methods
*Subjects – Is this a powered sample, or 36 participants in total were chosen by convenience? Please make this statement.*

**A power analysis has been added to PAGE 6: line 125 “Power analysis based on Beste et al. (2009; Fig. 2) showed an amplitude difference between PD and control groups of about 8 ±2 µV for both compatible and incompatible control trials for the P300 peak. With an alpha of 0.05 for a single comparison, a sample size of 18 per group yielded power of over 90%. “**

*Page 6, lines 120-122 – Please, cite the reference about clinical diagnosis of PD. In addition, were these participants diagnosed with idiopathic PD? Please, make it clear.*

**A reference about clinical diagnosis has been added to page 6; line 122. To clarify that all patients had idiopathic PD the following sentence was added to page 7: Line 144 “All patients had late onset (>55 years at time of diagnosis) idiopathic PD with a history of positive response to dopamine replacement therapy and no alterations in medication for 6 weeks prior to enrollment.”**

*Page 6, line 133 – How did the authors define “severe tremor”? Did the authors use any tools for that? Please, make it clear.*

**The term “severe” was replaced with “persistent,” referring to the fact that it was not well resolved with medication. Persistent tremor was defined as a score > 1 on items 16, 20 or 21 of UPDRS 3 evaluated on medication page 6: line 136).**

*Page 10, line 234 – Instead of citing the references 48, 54, please state the details that justify the use of uncorrected multiple comparisons.*

**We have edited this section (page 11; line 256-261) which now reads:** **Between-group analysis was corrected with a false discovery rate threshold of p<0.05. For the gamma band, we began with a conservative (multiple comparison corrected) threshold of p<0.05 as a first step, with a more liberal threshold (significant at p<0.05, uncorrected) as a second step if significant effects were not observed at a conservative level. The details of this approach have been described elsewhere [61,67,68]. As we have noted previously [61], the reduced power inherent to higher frequency oscillations often require us to rely on more liberal thresholds with a concomitant increase in risk for type 1 error [61,65].**

**The following statement has also been added to the limitations section (Page 27; line 633-638): “Despite our best efforts to reduce noise, for example enrolling only right-handed subjects with right side disease onset, gamma band activity was noisy, and the more stringent corrected p value yielded minimal consistent activation patterns. Thus our findings on gamma band activity have an increased susceptibility to Type 1 error, though no differences between PD and control participants were observed in the gamma band event related power changes.”**

Discussion

*I missed 3 important keys in this manuscript: statement of limitations, clinical relevance and conclusion. In regards of limitations, can the authors define any limitation in this study? I understand the importance of these results to understand how PD affects movement control and inhibition, but can the authors work out the clinical relevance of these findings?*

*Finally, a conclusion is indeed missing.*

**As suggested by the reviewer, sections for clinical relevance (Page 26; line 598), limitations (Page 27; line 626) and conclusions (Page 28; line 644) have been added to the manuscript.**

*Table 1. Is there any statistical between groups for age and gender? Regardless of statistical difference, please state the p-values in the text, or table.*

**Two sentences were added to the manuscript to provide this information (Page 7; line 142-144): “There were no differences across groups for age F(1, 35) = 0.65, p = 0.43. Gender distribution was not significantly different across groups X2(1, 35) = 2, p = 0.16.”**

*Did the authors use UPDRS total score? Please, present the part 3 scores as well. The authors mentioned in the discussion that no correlation was observed between UPDRS and cortical data. Have the authors observed any correlations using the motor score (part 3)? Why did the authors use UPDRS instead of MDS-UPDRS?*

**Both UPDRS total and part 3 are now included in the table. Correlation? The older version of the UPDRS was used because data collection began before the release of the NDS-UPDRS.**

*How was the dopamine equivalent calculated? Did authors use Levodopa equivalency daily dosage according to Tomlinson et al., 2010?*

**To clarify this point, the following statement was added (Page 7; line 151): “Dopamine equivalency for each PD participant’s daily medications was calculated based on conversion factors from Tomlinson et al., 2010.**

**Reviewer #3**: The authors' objectives were to examine PD-associated abnormalities in the magnitude and timing of oscillatory power in cortical networks subserving response activation and inhibition. The paper is interesting and contributes to the understanding of cortical networks subserving response activation and inhibition.

Major reviews:

*What do the authors call early Parkinson disease? Table 1 shows that some patients have more than 8 years of illness.*

**We agree that the word “early” is not accurate and it has been removed from the title and manuscript.**

*The hypothesis needs further explanation.*

**To better explain the rationale for our hypothesis, a paragraph on the underlying anatomy has been added to the introduction (Page 4; line 82-90). The introduction now summarizes existing research that motivated the anatomical, latency and frequency band power aspects of our hypothesis: “We tested the hypothesis that oscillatory activity in the frontal cortex subserving these functions had reduced power and delayed onset in PD compared to control participants.”**

*How many attempts at practice have been made? Did PD participants and control have the same practice time? Could this have influenced the task's performance during testing?*

**Training was minimal for both PD and control participants as the task was simple and easy to grasp. To clarify this point, we added details to the description of the training: “Subjects were trained prior to entering the scanner to respond only to the target arrow(s) with their index fingers pressing hand-held button boxes. Stimuli consisted of a series of stimuli that contained 2 trials of each trial type. All subjects executed this trial run once before scanning following which they reported understanding the task.” Page 7; line 159- page 8; line 165**

*Were patients evaluated ON or OFF? What does this interfere with the results?*

**To clarify that all study activities were preformed on medication, the statement about evaluation was changed to the following: “PD participants took their prescribed medication in the morning prior to study participation and performed the experiment in their best ON medication state.” (Page 7; line 146-148)**

**In addition, the following statement was added to indicate that the UPDRS was also performed on medication: “Participants with PD were given the Unified Parkinson Disease Rating Scale (UPDRS) on medication on a separate day from brain imaging.” (Page 6; line 138-139)**

**A statement about the impact of ON medication evaluation was added page 26; lines 601-603.**

*Were the conditions randomized or presented in blocks? Was the task learned during the experiment?*

**The statement, “Trial types were presented in random order” was added to end of the section describing the trials (Page 9; lines 195-196). Regarding learning, the task was very simple, and we did not observe an error pattern (fewer errors over time) that was indicative of learning.**

Discuss the implications of having found differences in cortical activity despite the same task performance.

**The discussion of the interpretation of differences in cortical activity with similar task performance have been expanded. The paragraph on the distribution of abnormal network activity in response to movement activation now includes a discussion of a pathological, compensatory and response strategy interpretation (Page 18; line 446). The possibility of compensation is also addressed in the discussion of frequency abnormalities (Page 25; line 565-579) and in the conclusions (page 28; line 645).**

A Conclusion section would be important.

**We agree, and a conclusions section has been added (Page 28; line 644).**

Minor review:

- *Place the units in the tables*

**Units have been placed in the tables**

- what is the patients' H&Y?

**Median H&Y has been added to Table 1**

- *are the values in table 3 reported as mean and standard deviation?*

**Yes, this information has been added to the legend.**

- *check the link to the page (*[*http://www.sourcesignal.com/dataeditor.html*](http://www.sourcesignal.com/dataeditor.html)*) that is not working*

**The link has been updated. The correct link is (**[**https://www.ctf.com/products**](https://nam01.safelinks.protection.outlook.com/?url=https%3A%2F%2Fwww.ctf.com%2Fproducts&data=04%7C01%7Cedisbr%40lsuhsc.edu%7C02a6bcf9c3ae4d9ce72408d8751392c7%7C3406368982d44e89a3281ab79cc58d9d%7C0%7C0%7C637388074212223647%7CUnknown%7CTWFpbGZsb3d8eyJWIjoiMC4wLjAwMDAiLCJQIjoiV2luMzIiLCJBTiI6Ik1haWwiLCJXVCI6Mn0%3D%7C1000&sdata=OHxczieREM6Evd%2FM0wZHP%2B7LTVayle1XCwi26uQ%2FHwM%3D&reserved=0)**).**

- *What was considered a wrong attempt?*

**An error was defined as a unilateral button press of the incorrect button, a unilateral response when a bilateral response was required or a bilateral response when a unilateral response was required. This definition was added to page 9, line 206.**
