# Supplementary material for: Cortical oscillatory dysfunction in Parkinson disease during movement activation and inhibition
Source: PLoS One. 2022 Mar 4;17(3):e0257711. doi: 10.1371/journal.pone.0257711 (PMC8896690; doi:10.1371/journal.pone.0257711)
Supplement: Attachment — Submitted filename: Response letter 06 04 21.docx [file pone.0257711.s002.docx]

We would like to thank the reviewers for their thoughtful comments on this manuscript. All suggestions have been incorporated into the latest version. Most notably, the discussion has been extensively edited based on reviewer comments to be more accessible. Specific responses are described below.

**Page 6: was there any inclusion and exclusion criteria for healthy controls? Please, state them.**

The inclusion and exclusion criteria for controls have been added on page 6, line 133.

**Page 6, end of last paragraph: “Using an alpha of 0.05 for a single comparison, a sample size of 18 per group yielded power of over 90%” What statistical test the authors refer to?**

The line now reads: Using an alpha of 0.05 for a single comparison, a sample size of 18 per group yielded power of over 90% for an independent, 2 sample t-test comparing PD and control groups. Similar results were obtained for a power analysis for latency measures.

**Page 12 end of last paragraph: “analysis of variance (ANOVA) with t-test post hoc analysis”. The authors should correct this to “… (ANOVA) with post hoc analysis using a p value of 0.05”**

The text has been changed as indicated.

**Caption for Table 3. Please, replace “#” with the word “number” or “amount”.**

The text has been changed as indicated.

**The authors first introduced the ROI in the results section. I suggest the authors introduce the regions of interest in the methods section.**

We have added the following sentence to the last paragraph of the introduction: “to measure anatomical, at the level of the cortical field region of interest, and physiological, specifically electrophysiological data.” A sentence about the ROI definition procedure and a reference have also been added to the methods on page 11, line 261. “Regions of interest (ROI) based on Brodmann nomenclature were derived from MNI coordinates in the normalized brain [68].”

**Why do the authors present p-values for only some of the differences, and not for all? Please, present p-values for all comparisons.**

The p values for the ANOVA’s were provided in the results while the p value for the NUTMEG analysis were provided in the methods, which, as indicated by the reviewer, was odd. The NUTMEG p values are now included in the results as well to clarify the statistics.

**Discussion – The discussion is too large (8 pages) and somewhat repeat the results. The authors have been comparing their results with literature rather than deeply discussing the meaning of the results found in the study. Please, update the discussion making it more concise and easier to follow.**

This comment was very helpful and the discussion has been extensively edited. The discussion has been reduced from just over 10 pages to 8 pages including additions suggested by reviewers. The discussion of previous work was reduced and put in context, making the discussion more concise and easier to follow. We also created a section on compensation (page 23, line 533) to improve the organization and flow of the discussion.

**Please see the manuscript from Pelicioni and colleagues (2020) where they discuss how people with PD exhibit reduced cognitive and motor cortical activity when undertaking complex stepping tasks requiring inhibitory control. Even though the authors have written the clinical implication in their most updated version of the manuscript, I still think the paper has many technical aspects and not enough clinical implications, and how these results can be translated to the understanding of motor impairment (such as inhibition impairments) in people with PD.**

We read the recommended reference with interest. The clinical significance section has been amended along the lines of this paper, including a reference to our work on neurorehabilitation of movement activation, and a modified statement about the clinical implications of inhibition deficits in PD. The end of the clinical implications section now reads: “Data on abnormal cortical activity may inform intervention strategies [156–158]. For example, cognitive neurorehabilitation targeting movement activation has shown some success [157,158]. Future studies of neurorehabilitation using MEG could yield clinically significant insight into the plasticity subserving recovery of function. Impairments of movement inhibition, though clearly present in PD, were described only recently [2] and, arguably, make subtle and complex contributions to classic motor signs such as gait or intricate stepping abnormalities [156]. As in most complicated diseases, the picture of Parkinson's disease becomes clearer with accumulation of evidence at all levels of the neural-cognitive system.”
